# Use of an Activity Monitor and GPS Device to Assess Community Activity and Participation in Transtibial Amputees

**DOI:** 10.3390/s140405845

**Published:** 2014-03-25

**Authors:** Brenton Hordacre, Christopher Barr, Maria Crotty

**Affiliations:** Department of Rehabilitation and Aged Care, Flinders University, Adelaide 5041, SA, Australia; E-Mails: chris.barr@flinders.edu.au (C.B.); maria.crotty@flinders.edu.au (M.C.)

**Keywords:** amputees, rehabilitation, technology, activity monitor, activity, participation, wearable sensors

## Abstract

This study characterized measures of community activity and participation of transtibial amputees based on combined data from separate accelerometer and GPS devices. The relationship between community activity and participation and standard clinical measures was assessed. Forty-seven participants were recruited (78% male, mean age 60.5 years). Participants wore the accelerometer and GPS devices for seven consecutive days. Data were linked to assess community activity (community based step counts) and community participation (number of community visits). Community activity and participation were compared across amputee *K*-level groups. Forty-six participants completed the study. On average each participant completed 16,645 (standard deviation (SD) 13,274) community steps and 16 (SD 10.9) community visits over seven days. There were differences between *K*-level groups for measures of community activity (F_(2,45)_ = 9.4, *p* < 0.001) and participation (F_(2,45)_ = 6.9, *p* = 0.002) with lower functioning K1/2 amputees demonstrating lower levels of community activity and participation than K3 and K4 amputees. There was no significant difference between K3 and K4 for community activity (*p* = 0.28) or participation (*p* = 0.43). This study demonstrated methodology to link accelerometer and GPS data to assess community activity and participation in a group of transtibial amputees. Differences in *K*-levels do not appear to accurately reflect actual community activity or participation in higher functioning transtibial amputees.

## 1. Introduction

Successful reintegration into the community following lower-limb amputation is a key aim of both rehabilitation clinicians and patients [1–4]. Community integration may be characterized by the domains of activity and participation as outlined in the International Classification of Functioning, Disability and Health (ICF) [5]. Prosthetic mobility is an activity which has been associated with improved quality of life [6], greater involvement in social activities [6,7] and activities of daily living [8,9], and is important for participation in employment and recreational roles [10]. Following amputation only 26%–62% of patients achieve outdoor mobility [11] limiting the capacity to participate in the community. For clinicians and researchers, assessment of community activity and participation following rehabilitation is a key marker of successful prosthetic rehabilitation and intervention effectiveness. Therefore there is a need to accurately quantify these domains.

Typically, the domains of activity and participation are assessed using either subjective or objective measures. Subjective measures, such as the Locomotor Capabilities Index [12] and activity diaries, have been shown to be unreliable and overestimate activity in lower-limb amputees [13,14]. Conversely, performance on objective clinical assessments have shown moderate to strong correlations with community based activity measures [15], and may be representative of the capacity to perform that activity in the community. Objective measures of ambulatory function are used by clinicians to guide prosthetic prescription and assess outcomes from rehabilitation. These measures include the amputee mobility predictor (AMP-PRO) [16], and timed walking tests of gait velocity (*i.e.*, 10 m walk test) and endurance (*i.e.*, 6 min walk test). Briefly, the AMP-PRO assesses a range of functional activities providing a score which assists in classifying amputee *K*-levels (K0, K1, K2, K3, K4) and guiding prosthetic prescription [17]. Higher *K*-levels (e.g., K4) indicate amputees with potential for greater functional ability, who may benefit from more advanced prosthetic componentry [16]. In addition to the AMP-PRO, clinically assessed timed walking tests of gait velocity and endurance are often used by clinicians. These tests are associated with community activity and represent complementary measures to further interpret community activity and participation potential [15,18–20]. However, a limitation of clinical assessments is that they fail to replicate the range of physical demands and unpredictable nature of community ambulation and participation, and as such, these measures may not accurately reflect actual levels of community activity and participation [1].

With recent advances in wearable technology [21], accelerometer-based monitoring devices to assess step-counts and global positioning satellite (GPS) devices to assess location offer the ability to obtain accurate objective measures of community activity and participation [21–26]. Activity data derived from accelerometer devices has been successfully collected in amputees previously [13,15,27], but, by its nature, does not provide information about the location in the community where the activity occurs. The addition of GPS data provides a means of mapping position in the community, but introduces challenges for ensuring reliable data capture and synchronisation with activity data [28]. Recently, GPS and step count data were collected in a transfemoral amputee case study, demonstrating potential to provide accurate information for clinicians [29]. However, it is currently unknown if separate accelerometer and GPS data can be captured simultaneously and successfully linked in order to assess community activity and participation in a larger cohort of transtibial amputees. Additionally, we are unaware of any studies which have used separate wearable technology devices to determine if differences do exist for community activity and participation between amputee *K*-levels. We believe it is important for the field of amputee rehabilitation to further investigate the use of wearable technology to assess community activity and participation, and determine the nature of relationships between community and clinic based measures.

### Aims/Hypothesis

The primary aim of this study was to assess the ability to use wearable technology (accelerometer-based monitoring devices to assess step-counts and GPS devices) to measure community activity and participation in rehabilitated transtibial amputees. The secondary aim was to determine if community activity and participation was different for predicted *K*-levels as assessed by AMP-PRO and timed mobility measures. We hypothesised that the combination of accelerometer and GPS data to assess community activity and participation would be feasible and that community based measures would differ between amputee *K*-levels.

## 2. Methods

### 2.1. Participants

Forty seven rehabilitated unilateral transtibial amputees were recruited. All participants had been correctly fitted with a definitive prosthesis at least six months prior to testing, and correct prosthetic fit and comfort were confirmed with the participant and their prosthetist prior to inclusion in the study. Participants were eligible if they achieved prosthetic mobility. Amputees not provided with a prosthesis for mobility (functional level K0) were excluded. The majority of recruited participants were male (79%), with a mean age of 59.7 (range 19–98) years and were 16.2 (standard deviation (SD) 18.9) years since amputation. Primary indications for amputation were trauma (38%) or peripheral vascular disease (38%). Ethical approval was provided by the Southern Adelaide Clinical Human Research ethics committee and all participants provided written informed consent in accordance with the Declaration of Helsinki.

### 2.2. Equipment

#### 2.2.1. Step Activity Monitor

A StepWatch3 Activity Monitor (SAM) (Cyma Corp, Seattle, WA, USA) was fitted to each participant's prosthesis in accordance with manufacturer's recommendations. The SAM is an accelerometer and microprocessor based activity monitor measuring 6.5 cm × 5.0 cm × 1.5 cm verified for use in people with lower-limb amputations [30]. The SAM was set to record stride count data for each minute of programmed use [15]. Step count data was obtained by multiplying the stride count by two. Data from the SAM was downloaded using StepWatch software (version 3.1b), and stored within the software database.

#### 2.2.2. Global Positioning System

A QStarz BT-Q1000XT (Qstarz International Co., Ltd., Taipei, Taiwan) 66-channel tracking global positioning system (GPS) travel recorder was used to record latitude, longitude, local date and time of each participant's position for every five seconds of programmed use. The device measures 7.2 cm × 4.7 cm × 2.0 cm, has a battery life of 42 h and accuracy error of less than three metres. Data from the GPS unit were imported to QTravel software (version 1.46) and stored within the software database.

### 2.3. Procedure

**Figure 1.**
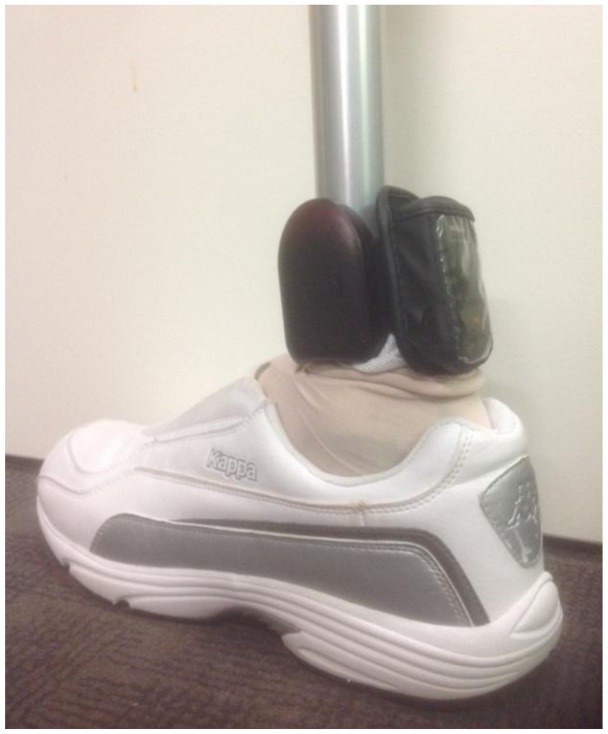
The SAM and GPS devices attached to a prosthesis for data collection. The SAM was positioned according to the manufacturers' recommendations. The GPS was attached to the same strap as the SAM device for convenience.

Participants were supplied with SAM and GPS devices. Both devices were secured to the participant's prosthesis with a single Velcro strap (see Figure 1). The devices remained attached to the prosthesis for the duration of the study period. The SAM and GPS devices were programmed for data collection using separate networked computers, thereby ensuring local time for each device was identical and preventing mismatched times in the data linkage process. Participants wore the SAM and GPS devices for a period of seven consecutive days and were supplied with a battery charger and clear written instructions for charging the GPS device nightly [31]. At the time of provision of SAM and GPS devices, clinical characteristics (age, time since amputation and indication for amputation), employment status and standard clinical measures were collected. These standard clinical measures were the AMP-PRO, gait velocity, and gait endurance assessed by the six minute walk test (6MWT). *K*-levels were assigned by an experienced physiotherapist during assessment of standard clinical measures and clinical characteristics, primarily using the AMP-PRO to guide classification [16]. Briefly, K1 are limited or unlimited household ambulators, K2 are limited community ambulators, K3 are community ambulators capable of traversing most environmental barriers, and K4 amputees are capable of high impact, stress or energy levels [16]. Assessment of gait velocity and the 6MWT were selected based on their previous use in lower-limb amputees [16,18,19]. Gait velocity was assessed using an instrumented GAITRite walkway (CIR-Systems Inc., Sparta, NJ, USA) with embedded pressure sensors to capture individual footfall data over an active area of 4.9 m × 0.6 m. Participants completed 10 consecutive walking trials over the GAITRite at their self-selected gait speed. Data were collected at 120 Hz and analysed using GAITRite software (version 4.5). The 6MWT [32] was used to assess endurance and was assessed over a 20 m track with the participant turning at each end.

### 2.4. Data Analysis

#### 2.4.1. Data Linkage

Datasets obtained from the SAM and GPS devices were exported as comma-separated value (CSV) files from the respective software for each device. Within Microsoft Excel (2010) both SAM and GPS datasets were ordered chronologically and trimmed to ensure each dataset contained only seven consecutive complete periods of 24 h. As the SAM data was recorded per minute and the GPS per five seconds, local time of recorded data for both the SAM and GPS was trimmed to hour and minute values only so that the two sets of time values were identical. Local time for each dataset was converted to a time value ranging from 0 to 0.99930556, representing unique time values from 00:00 to 23:59. In a similar manner local date was converted to a date value represented as an integer for dates ranging from 1 January 1900 to 31 December 9999. A variable coded as Time_Date was generated in both datasets as a unique local time and date identifier and was calculated as the addition of the time value and date value. Latitude and longitude data obtained from the GPS was linked to step count data using Microsoft Excel's “lookup” function to link the unique Time_Date variable created. Similar use of date and time stamps has been reported in previous studies to link data from various forms of wearable technology [33,34]. As a result of reducing GPS local time data to hour and minute values, up to 12 GPS latitude and longitude values were available for each minute of recorded data, and in this instance, the first latitude and longitude data values were used to link to the SAM dataset. The final linked dataset therefore contained step count and GPS latitude and longitude data for each minute of the seven consecutive days of data collection. Community visits were defined as events where the participant left their home and attended a location in the community [5]. Individual community visits were analysed by recounting latitude and longitude data for the assessed seven consecutive day period in chronological order within QTravel (version 1.46). QTravel incorporates Google Maps and Google Earth software which utilises satellite imagery to provide geographic information. Community visit events were visually identified from this geographic information. These events were then manually coded as one of seven community participation categories external to the participants home (see Box 1 for a description of categories). If required verbal confirmation was obtained from participants ensuring accurate identification of community participation. Community participation, defined as involvement in life situations [5], was assessed as the total number of individual visits to these categories. Community activity was assessed as the total step count out of home and was calculated as the sum of step counts across the seven community participation categories [5,35].

Box 1. **The seven community categories with examples which were used to assess community activity and participation in rehabilitated transtibial amputees**

**Community Participation Categories****Category****Examples**EmploymentPaid employment activitiesResidentialHousing other than own homeCommercialShopping centres, local shopsHealth servicesHospital, general practitioner, physiotherapist, chiropractor, pharmacistRecreationalOval, sports, beach, walk in communitySocialRestaurant, café, hotel, cinema

#### 2.4.2. Statistical Analysis

The normality of data was checked with a Shapiro-Wilk normality test and where required data transformations were performed to achieve normality. To assess completeness and quality of GPS data we assessed missing GPS data points (%) prior to linkage, and missing step count data (%) in the linked dataset. Missing GPS data were assessed by comparing the expected number of cells with recorded data (*n* = 120,960) to observed number of cells with recorded data. Missing step count data from the linked datasets were analysed as the difference between step counts linked to GPS data and total step counts from the SAM. Descriptive statistics were used to characterise community activity based step counts and community participation visit data for each of the community participation categories. Amputee *K*-levels were analysed as three separate groups due to the low number of recruited amputees in the K1 and K2 categories. The categories were K1/2, K3, and K4. Clinical characteristics of age and time since amputation were analysed between *K*-level groups using separate one-way ANOVA's. Employment status and indication for amputation (peripheral vascular disease (PVD), trauma, other) were analysed between *K*-level groups with a Chi-Square test. Separate one-way ANOVA's were used to determine if there were differences in community based step counts, community visits, gait velocity, and the 6MWT. Post-hoc analyses were performed for ANOVA analyses with significant results using Bonferroni adjustment. Pearson correlation was used to assess the association between total step count from the accelerometer data alone and our measure of community activity and participation to determine if an accelerometer alone could be used as a simple assessment of community activity and participation. Significance level was set at *p* ≤ 0.05 and SPSS software was used for all statistical analyses (IBM corp. Released 2010. IBM SPSS Statistics for Windows, Version 19.0).

## 3. Results

A total of 47 transtibial amputees were recruited to participate in the study. One was excluded due incomplete GPS data resulting from failure to charge the GPS battery as instructed. For the remaining 46 datasets, 6.5% (SD 7.3%) of GPS data was unavailable due to lost signal. As a result of incomplete GPS data due to signal loss, 5.3% (SD 5.9%) of all steps recorded by the SAM were not linked to GPS positional data. For our measures of community activity and participation, amputees completed on average 16,645 (SD 13,274) steps in the community and visited 16.4 (SD 10.9) community facilities over a consecutive seven day period. A summary of activity and participation measures is provided in Table 1.

Clinical characteristics (age, time since amputation and indication for amputation) and employment status were analysed for *K*-level categories. There were significant differences between *K*-level categories for age (F_(2,43)_ = 4.2, *p* = 0.022), but there was no significant difference for time since amputation (*p* = 0.104), indication for amputation (*p* = 0.112) or employment status (*p* = 0.077). Post-hoc analysis revealed K1/2 amputees were older than K4 amputees (95%CI 4.8–29.9, *p* = 0.008). There was no significant difference in age between K1/2 and K3 amputees (*p* = 0.098), or between K3 and K4 amputees (*p* = 0.171) (see Table 2).

There were differences between *K*-level groups for our defined measures of community activity (F_(2,43)_ = 9.4, *p* < 0.001) and community participation (F_(2,43)_ = 6.9, *p* = 0.002). As expected, post-hoc analysis revealed K1/2 amputees completed significantly less community steps than K3 amputees (95%CI 8.2–128.3, *p* = 0.021) and K4 amputees (95%CI 39.2–150.0, *p* < 0.001). However, there was no significant difference in the number of community steps between K3 amputees and K4 amputees (*p* = 0.283). For community participation, post-hoc analysis revealed K1/2 amputees were involved in significantly less community visits than K3 amputees (95%CI 0.01–0.64, *p* = 0.049) and K4 amputees (95%CI 0.14–0.74, *p* = 0.002). However, there was no significant difference in the number of community visits undertaken by K3 and K4 amputees (*p* = 0.431).

There were differences between *K*-level groups for gait velocity (F_(2,43)_ = 28.5, *p* < 0.001) and 6MWT distance (F_(2,43)_ = 22.8, *p* < 0.001). Post-hoc analysis revealed K1/2 amputees had significantly lower gait velocity than K3 amputees (95%CI 0.11–0.60, *p* = 0.002) and K4 amputees (95%CI 0.40–0.85, *p* < 0.001). Amputees categorised as K3 had significantly lower gait velocity than K4 amputees (95%CI 0.11–0.42, *p* < 0.001). For the 6MWT, post-hoc analysis revealed K1/2 amputees walked significantly less distance than K4 amputees (95%CI 119.6–309.1, *p* < 0.001). Amputees categorised as K3 walked significantly less distance than K4 amputees (95%CI 62.2–193.2, *p* < 0.001). There was no difference between K1/2 and K3 amputees (*p* = 0.125). A summary of community and clinic based measures for each *K*-level group is provided in Table 3.

**Figure 2.**
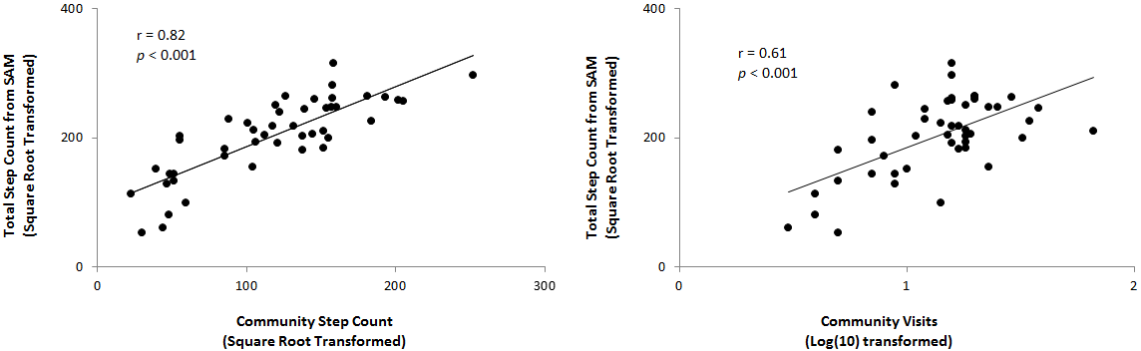
Pearson correlation analysis of total step count (obtained from the SAM) over a seven day period and our measure of community activity (**left**) and community participation (**right**). In both instances there was a significant positive correlation indicating that activity data from a single accelerometer device (e.g., SAM) may provide good indication of community activity and participation.

There was a significant positive correlation between our defined measure of community activity (community based step counts) and total step counts obtained from the SAM alone (*r* = 0.82, *p* < 0.001). There was also a significant positive correlation between our defined measure of community participation (number of community visits) and total step counts obtained from the SAM alone (*r* = 0.61, *p* < 0.001) (see Figure 2).

## 4. Discussion

This study investigated community activity and participation of transtibial amputees using a simple approach to linking data recorded from separate commercially available accelerometer and GPS devices. Findings from this study demonstrate that data collected from a combination of accelerometer and GPS devices is feasible with a patient group over a seven day period, and the data obtained from these devices had limited interruption due to inadequate GPS signal.

Amputee participants were below the recommended daily step counts for healthy adults, and fall into the category of “low activity” [36]. Additionally this study showed that K3 and K4 transtibial amputees demonstrated a wide range of actual community activity and participation levels, which likely contributed to non-significant statistical difference between these functional categories for the measures of community activity and participation. These findings are in contrast to clinically assessed measures of gait velocity and endurance (6MWT) which were able to differentiate the K3 and K4 functional categories. This suggests that while K4 transtibial amputees have the functional capacity to perform at a higher level in the community, in practice many do not achieve levels of community activity and participation greater than that of K3 amputees.

Community activity and participation are key indicators of successful rehabilitation and intervention effectiveness [1,5]. Recent advances in wearable technology have provided rehabilitation clinicians easier access to means of quantifying measures of activity and participation for a range of applications [21,22,37–42]. For amputee rehabilitation, wearable technology may be clinically appropriate to aid prosthetic prescription and to guide rehabilitation interventions. Similar data has been used previously in a case study [29] and we believe this to be the first study to report linked accelerometer and GPS data in a cohort of transtibial amputees. The methodology presented here may be transferable to other patient populations. The accuracy of wearable GPS devices integrated with geographic information allowed recording of a range of categorised community participation events. Linked SAM and GPS data provided an opportunity to analyse step counts in these categorised locations, and in the community in general, as a measure of community activity. Here we were able to demonstrate amputees performed the majority of activity within the home. For community based activity, the majority was performed in the work place for those amputees who were employed, and the most common community participation was visiting commercial facilities (shopping centres and local shops). The selection of participation events was based on typical activities of amputees, but could be modified and adapted for various patient populations as required. Although GPS devices have potential use in rehabilitation and research of clinical populations there are technical limitations. Primarily, GPS devices rely on satellite signal and have limited capacity for indoor use [28]. Data from this study demonstrates that a small proportion (6.5%) of data recording was lost from the GPS data due to insufficient satellite signal which occurred as a result of monitoring everyday activities in this cohort of amputees. This small proportion compares well to previous activity monitoring studies in stroke which report acceptable GPS data loss of 13% [43].

To our knowledge this to be the first study to report community activity and participation from linked accelerometer and GPS data in a transtibial amputee population. This study defined community activity and participation as community based step counts and community visits based of previous literature [5,35]. From linked SAM and GPS data we were able to demonstrate that amputees categorised as K1 or K2 performed at lower community activity and participation levels than amputees categorised as either K3 or K4. This finding is not unexpected given that these higher functioning amputees had higher rates of employment. Interestingly there was no statistical difference in community activity and participation between amputees categorised as K3 and K4, most likely due to the wide range of community activity and participation levels within these two groups. This may be surprising given the discrepancies in employment status between these two groups. Objective clinical measures also confirm that K4 amputees do indeed have the functional capacity to perform at higher levels than K3 amputees [15,16]. A potential reason for this lack of significant difference in community activity and participation between K3 and K4 amputees relates to properties of this assessment. There are no subcategories within each *K* level. Therefore the difference between a high level K3 and low level K4 are likely to be minimal. Conversely, the differences between a low K3 and a high K4 may be quite large in comparison. Additionally, this system was developed to predict prosthetic requirements of amputees based on their likely requirements in the community. These categories provide indication of the most suitable prosthetic componentry, and do not reflect actual performance. Therefore, these findings may indicate that the predictive K level categories may not reflect actual community activity and participation levels.

Previous literature suggests age [44–48], comorbidities [44,46] and indication for amputation [46] to be factors potentially limiting amputees to perform functionally in the community. Differences in age were found between the K1/2 and K4 amputees. We would expect to find these differences as previous studies indicate that lower functioning amputees are typically older [49]. Therefore the functional differences between K1/2 and K4 amputees may be a combination of lower functional abilities and older age. However, we were unable to demonstrate difference between K3 and K4 amputees for both age and indication for amputation suggesting these factors have not limited performance of K4 amputees in the community. Further research in a larger cohort of amputees should be undertaken to decipher implications of these findings and investigate why amputees categorised as K3 and K4 perform functionally similar in terms of community activity and participation. Clinical implications of this study suggest higher functioning amputees, categorised as K3 and K4, do not achieve the recommended daily step counts required for active lifestyles [36]. These higher functioning amputees may require objective community measures (such as accelerometers of GPS devices) to more accurately determine differences in functional abilities, rather than commonly adopted clinical measures (*i.e.*, gait velocity and endurance). While the combination of separate accelerometer and GPS devices may present difficulties in linking the data and interpretation clinically, we demonstrated positive correlations between accelerometer data (total step count) on its own obtained from the SAM and our measures of community activity and participation. Hence, a simple accelerometer device to assess step-count activity may be an appropriate assessment to differentiate amputees' categorised as K3 and K4 and should be considered for clinical use.

### Study Limitations

There are several limitations to this study which should be acknowledged. First, due to lost GPS signal, 5.3% of step count data was lost in the data linkage procedure. Although this percentage is low, it may represent a potential bias in this procedure and subsequent data analysis. Second, the data linkage procedure may limit the clinical usefulness of this methodology. However, we have shown a high correlation with total step count and our measure of community activity, and a moderate correlation with total step count and our measure of community participation. Third, we acknowledge the measure of community participation adopted may not completely encompass all aspects of community participation as described by the ICF [5]. For example, employment, social and recreational roles may be fulfilled from within a person's home, and therefore the exclusion of this data may not accurately represent community participation. However, a recent review summarised community participation as involvement in activities that occur outside the home, or involve a non-domestic nature [50]. We also suggest that participation in activities outside the home present greater mobility and social challenges, and are likely to represent greater community integration. Fourth, this study may also have benefited from inclusion of a participant diary to record daily community activity and participation. Although participation events were confirmed verbally with participants where required, it has previously been reported that a diary should be included in these studies to aid confirmation of activities, despite accuracy of diary recorded information being inferior to GPS recorded data [13,43]. Inclusion of a travel diary may have aided information recall in these instances. Fifth, results of this study with unilateral transtibial amputees may not be generalisable to other amputee populations, and further investigation is required to determine activity and participation of individuals with different levels of amputation; Finally, amputees recruited to participate in this study were primarily higher functioning (K3 and K4), with a relatively high percentage of traumatic amputees, and lower percentage of vascular amputees compared to that normally found in prosthetic rehabilitation services [51]. We suggest this is likely due to the long post-operative period in this study, and the poor mortality rates following rehabilitation for vascular amputees [52]. These results may not accurately reflect lower functioning K1 amputees, or more recent amputees. Despite this, we still observed relatively low activity levels amongst recruited amputees.

## 5. Conclusions

This study demonstrated a simple methodology to link step count and GPS data to assess community activity and participation in a group of unilateral transtibial amputees. We found amputees predicted to be higher functioning performed better in clinical assessments of gait velocity and endurance. However, they exhibited a wide range of community activity and participation levels. Therefore differences in *K*-levels may not accurately predict actual community activity or participation in higher functioning transtibial amputees. Step count data obtained from the SAM was shown to have a high correlation with community activity and participation and may be a good clinical tool to stratify higher functioning amputees based on actual activity as opposed to predicted activity.

## Figures and Tables

**Table 1.** A summary of community activity and participation for each participation category.

**Activity**	**Step Count Mean (SD) per Week**	**Community Visit Mean (SD) per Week**
Employment	5,323 (11,873)	3.4 (8.4)
Residential	2,603 (3,165)	2.9 (2.8)
Commercial	3,909 (4,102)	5.2 (3.7)
Health Service	776 (1,280)	1.1 (1.4)
Recreational	1,950 (3,604)	1.0 (1.5)
Social	1,733 (3,512)	1.9 (1.7)
Other	350 (723)	0.8 (1.5)
Home	25,285 (14,366)	-
Lost in linkage	2,353 (3,077)	-
Unidentified	222 (612)	-
Total	44,504 (22,600)	16.4 (10.9)

Lost in linkage = step count data that was recorded on the SAM while there was inadequate satellite signal for the GPS device; Unidentified = step count data that was unable to be categorised as one of the seven community participation categories, or home.

**Table 2.** Clinical characteristics for *K*-level categories.

	**K1/2 (*n* = 5)**	**K3 (*n* = 13)**	**K4 (*n* = 28)**	**Statistic**
Age (years), mean (SD)	74.2 (14.8)	62.9 (16.8)	57.1 (9.8)	*p* = 0.022
Time since amputation (years), mean (SD)	5.8 (8.5)	9.7 (13.9)	20.7 (21.2)	*p* = 0.104
Indication, n (%)				*p* = 0.125
PVD	4 (8.7%)	7 (15.2%)	7 (15.2%)	
Trauma	1 (2.2%)	4 (8.7%)	14 (30.4%)	
Other	0 (0%)	2 (4.3%)	7 (15.2%)	
Employed, n (%)	0 (0%)	3 (23%)	13 (46%)	*p* = 0.077

PVD, peripheral vascular disease; other indications for amputation include congenital, infection and tumor.

**Table 3.** Mean (SD) community and clinical measures for *K*-level categories.

	**K1/2 (*n* = 5)**	**K3 (*n* = 13)**	**K4 (*n* = 28)**
Community Step Count	1,379 (1,012)	14,483 (16,585)	19,463 (11,016)
Community Visit	7.2 (4.3)	13.77 (5.8)	19.32 (12.4)
Gait Velocity (m/s)	0.65 (0.2)	1.01 (0.2)	1.28 (0.2)
6MWT (m)	212.0 (79.4)	298.6 (74.5)	426.3 (79.8)

6MWT, six minute walk test.
